# CaSiO_3_-HAp Structural Bioceramic by Sol-Gel and SPS-RS Techniques: Bacteria Test Assessment

**DOI:** 10.3390/jfb11020041

**Published:** 2020-06-12

**Authors:** Evgeniy Papynov, Oleg Shichalin, Igor Buravlev, Anton Belov, Arseniy Portnyagin, Vitaliy Mayorov, Evgeniy Merkulov, Taisiya Kaidalova, Yulia Skurikhina, Vyacheslav Turkutyukov, Alexander Fedorets, Vladimir Apanasevich

**Affiliations:** 1 Institute of Chemistry, Far Eastern Branch of Russian Academy of Sciences, 159, Prosp. 100-letiya Vladivostoka, Vladivostok 690022, Russia; oleg_shich@mail.ru (O.S.); buravlev.i@gmail.com (I.B.); nefryty@gmail.com (A.B.); arsuha@gmail.com (A.P.); 024205@inbox.ru (V.M.); merkulov@ich.dvo.ru (E.M.); kaydalova@ich.dvo.ru (T.K.); 2 Far Eastern Federal University, 8, Sukhanova St., Vladivostok 690091, Russia; fedorets.alexander@gmail.com; 3 Pacific State Medical University, 2, Ostryakov Aven., Vladivostok 690990, Russia; eesku@mail.ru (Y.S.); vyach.12593@mail.ru (V.T.); oncolog222@gmail.com (V.A.)

**Keywords:** porous bioceramics, wollastonite, hydroxyapatite, sol-gel technology, spark plasma sintering–reactive synthesis, bacterial test

## Abstract

The article presents an original way of getting porous and mechanically strong CaSiO_3_-HAp ceramics, which is highly desirable for bone-ceramic implants in bone restoration surgery. The method combines wet and solid-phase approaches of inorganic synthesis: sol-gel (template) technology to produce the amorphous xonotlite (Ca_6_Si_6_O_17_·2OH) as the raw material, followed by its spark plasma sintering–reactive synthesis (SPS-RS) into ceramics. Formation of both crystalline wollastonite (CaSiO_3_) and hydroxyapatite (Ca_10_(PO_4_)_6_(OH)_2_) occurs “in situ” under SPS conditions, which is the main novelty of the method, due to combining the solid-phase transitions of the amorphous xonotlite with the chemical reaction within the powder mixture between CaO and CaHPO_4_. Formation of pristine HAp and its composite derivative with wollastonite was studied by means of TGA and XRD with the temperatures of the “in situ” interactions also determined. A facile route to tailor a macroporous structure is suggested, with polymer (siloxane-acrylate latex) and carbon (fibers and powder) fillers being used as the pore-forming templates. Microbial tests were carried out to reveal the morphological features of the bacterial film *Pseudomonas aeruginosa* that formed on the surface of the ceramics, depending on the content of HAp (0, 20, and 50 wt%).

## 1. Introduction

Sustainable development of modern biotechnology in the field of regenerative and reconstructive bone surgery is governed by the quality of the available biomaterials, a specific class of systems with a unique set of physico-chemical and mechanical characteristics (chemical inertness, microstructural diversity, mechanical strength, fracture resistance, and durability), as well as biocompatibility (non-toxicity, bio-inducivity, bio-conductivity, and bio-resistivity) [1,2]. The combination of such properties in one product is a challenging scientific and technological problem in the search for affordable raw materials and simple processing technologies to fabricate the final products.

Calcium monosilicate, β-wollastonite (CaSiO_3_), is actively studied at the moment due to its applications in traumatology, orthopedics, dentistry, maxillofacial surgery, and other areas of medicine for the recovery, replacement, and reconstruction of the damaged tissue in a living organism [1,3,4,5,6,7,8,9,10,11,12,13,14]. Owing to β-wollastonite’s ability to activate the growth of the apatite layer on its surface due to the pronounced osteoconduction and bio-resorption via exchange of Ca^2+^ and SiO_3_^2−^ with the bioorganic medium, the material is promising as an artificial bone substitute [15,16]. In terms of chemical bonding, wollastonite ceramic is close to the inorganic bone matrix and has no toxic effect on the body. In addition, it is corrosion-resistant, thermally stable, and chemically inert or bioactive under long-term exposure in bioorganic environments [17]. In order to achieve maximum similarity to the functional and structural parameters of the bone tissue, β-wollastonite can be modified with hydroxyapatite (HAp), which is a complete analogue of a living bone [14,18,19].

Chemical composition is an important, but not the only characteristic, of bioceramics implants. Their uniqueness to a large extent is determined by a combination of structural and mechanical characteristics. Porous and mechanically stable ceramics represents a model of cellular “spongiose” material that serve as a matrix for ingrowth (implantation) of the bone tissue during osteointegration, a process of recovering lost tissular structures in the living organism in the presence of the implant. Osteointegration intensity depends on pores presented in the implant, particularly, on their size, quantity, and interconnectivity. The bio-integration process, which is based on the reproduction of osteogenic cells, requires large macropores, sized 100–135 µm, as well as on the thin submicron and nanosized pores that are commensurate with blood plasma proteins for their effective adsorption. Stability of such a matrix has to be optimal for the uniform distribution of mechanical load between natural and artificial bone that avoids the possibility of excess decomposition of the bone tissue [20,21]. Thus, it is obvious that load-resistant ceramics with a hierarchical pore size distribution is necessary for practical medicine.

From an implant manufacturing standpoint, fabrication of the β-wollastonite powder and its composites with the HAp-required characteristics and properties is not difficult. This can be done by sol-gel, hydrothermal, and precipitative synthesis technologies [6,22,23,24,25,26,27,28,29,30]. These approaches are easy to implement and allow to vary the size and shape of the crystallites and to tailor the surface morphology. On the contrary, to manufacture the bulk ceramics of the required geometry, characteristics, and properties is far more challenging due to the rigid thermal conditions of the powder treatment during consolidation into dense ceramics. During the fabrication of wollastonite ceramics, most of the conventional sintering methods do not provide the preservation of the porous structure of the wollastonite [31] due to the negative impact of the heat treatment on the composition and structure of the final product. The reason lies in the phase instability of the HAp above 1000 °C, as well as the destruction of the porous volume and the activation of grain growth, which leads to distortion or destruction of the porous frame in the solid body and negatively affects the final properties of the biocomposite [14,32,33].

The problems in the synthesis of porous ceramics described above are solved by using the technology of spark plasma sintering (SPS) [34,35,36,37,38]. The unique mechanism of powder consolidation in this technology is based on the spark plasma current flowing through the sample under pressure, which provides a number of advantages over traditional methods, because in this case ceramic wollastonite with a tailored microstructure and exceptional mechanical characteristics is rapidly formed [39,40,41]. The structural strength of the SPS ceramics is achieved without the need for additional reinforcement components that contaminate the final product. Our early studies have identified the above-described prospects of SPS application for the synthesis of nanostructured bioceramic wollastonite [42,43,44,45] with its bioactive properties being assessed “in vivo” [46]. Additionally, these studies showed several original methods for developing a porous structure of the ceramics that is similar to the texture of bone tissue by introducing various porous templates. However, the technology of spark plasma sintering–reactive synthesis (SPS-RS) should be considered even more promising for the production of innovative ceramics. SPS-RS is based on the chemical interaction between the starting components of the sintering mixture under the influence of spark plasma, resulting in a new type of the final product [47,48,49]. This approach allows one to directly obtain different materials with unique properties based on multi-component ceramic systems. The literature substantiates the advantages of SPS-RS compared to conventional SPS and hot pressing [41]. SPS-RS efficiency for bioceramic synthesis is based on the local character of the chemical interaction between the components of the reaction mixture (resulting in biocomponents) under the spark plasma heating, taking place on the interparticle contacts.

Such local heating favors the formation of the fine-crystal phases of the biocomponent, which will be more biologically compatible or resorbed depending on its chemical composition. Additionally, microlocal heating allows the reactions to proceed at lower temperatures; thus, not disrupting the metastability of the substances and increasing the limit of their thermodestruction. We have recently explored the feasibility of this approach for fabrication of ZrO_2_ ceramics containing the bioactive phosphate compounds obtained “in situ” under SPS-RS conditions [50]. For the synthesis of ceramic HAp-containing wollastonite, similar studies have not been conducted before.

In this regard, the work intends to study the way to obtain a crystalline ceramic HAp–wollastonite composite via solid phase transformation of amorphous xonotlite and “in situ” interaction of the reaction mixture (CaO and CaHPO_4_) under SPS conditions. Additionally, the way to tailor the porous structure of the ceramics using pore-forming templates has been investigated. Microbial tests were conducted to assess the possible risks of an infectious process caused by bacterial contamination of the ceramics.

The proposed non-standard SPS-RS approach can pave the way to fabrication of biocompatible ceramics for bone tissue engineering; thus, contributing another flexible strategy for the synthesis of biomaterials.

## 2. Materials and Methods

### 2.1. Materials

Sodium metasilicate (Na_2_SiO_3_·5H_2_O) and calcium chloride (CaCl_2_·2H_2_O) have been used as the main precursors for calcium silicate synthesis. Calcium oxide (CaO) and calcium hydro-orthophosphate (CaHPO_4_) were used to synthesize HAp. All reagents are of 99.98% purity (Sigma-Aldrich, St. Louis, MO, USA). Siloxane acrylate latex “KE 13–36” (LLC “Astrokhim”, Electrostal, Russia, solid phase content 50%, average particle size 160 [51,52]), carbon fiber (CF) “AUT-M” (TS 1916-346-04838763-2009, ENPO “Inorganica”, Electrostal, LTD Russia), and a graphite powder (GP) fraction 1–5 µm (GOST 7885-86, “Khazar”, Turkmenistan) were used as the pore-forming templates.

### 2.2. Synthesis Technique

The composite CaSiO_3_ (wollastonite)/HAp ceramics was prepared in three successive stages. Initially, the method of sol-gel (template) synthesis yielded a composite material (xerogel) based on hydrated calcium silica (xonotlite, Ca_6_Si_6_O_17_·2OH) mixed with polymer latex (pore-forming template). Then, a sintering mixture (SM) was made by blending the obtained xerogel with the reaction mixture components (RM) for the formation of HAp. Finally, the consolidation of SM using SPS technology was carried out.

#### 2.2.1. The Sol-Gel (Template) Synthesis of Amorphous Composite Powder Ca_6_Si_6_O_17_·2OH (Xonotlite)

A total of 50 mL of 1.0 M calcium chloride solution and 50 mL of 1.0 M sodium metasilicate solution were added batchwise to 150 mL of a siloxane–acrylate water solution (latex:water ratio 1:30) under intense stirring. Then, the solution was stirred for three hours at 100 °C until a dense gel was formed, which after boiling was cooled to room temperature (25 °C). After that, 16.6 mL of a 1.0 M calcium chloride solution and 10 mL of 1.0 M ammonium hydrophosphate were added to the obtained gel and stirred for 1 h at room temperature (25 °C). The resulting gel was filtered and washed with distilled water until a negative reaction to the chloride ions was obtained, and then dried for about 5 h at 90 °C.

Fabrication of wollastonite proceeded according to the chemical reaction below:

CaCl_2_ + Na_2_SiO_3_ → CaSiO_3_ + 2NaCl (1)

#### 2.2.2. Preparation of the Sintering Mixture Added with Reaction Mixture Additive

The composite xerogel obtained according to Section 2.2.1 was mixed with the components of the reaction mixture (RM) CaO+CaHPO_4_ at the planetary mill at a rotation rate of 870 rpm for three cycles for 15 min. Carbon fiber and powder graphite (Table 1) were added into this mixture as additional pore-forming agents during ball milling.

Reaction of HAp formation:

CaO + CaHPO_4_ → Ca_10_(PO_4_)_6_(OH)_2_ + 2H_2_O (2)

#### 2.2.3. SPS-RS Fabrication of the Composite CaSiO_3_ (Wollastonite)/HAp Ceramics

The SM powder (fraction 0.1–0.5 mm), obtained via ball milling (see Section 2.2.2), was put into a graphite die (outer diameter—30 mm, internal diameter—15.5 mm, and height—30 mm), prepressed (20.7 MPa), and then the green body was put into a vacuum chamber (pressure 6 Pa) and sintered on a LABOX-625 SPS machine “Sinter Land Incorporation, LTD” (Niigata, Japan). Sintering was executed at 900 °C at a heating rate of 100 °C/min and 5 min holding at maximal temperature. After sintering, the samples were cooled down to room temperature (25 °C) for 40 min. Pressure loaded onto the sample during sintering was 24.5 MPa and remained constant throughout the process. The frequency of the low-voltage pulse generated in the On/Off mode was 12/2, with a pulse packet duration of 39.6 ms and a 6.6 ms pause between the pulses. The maximum current and voltage during sintering were 500 A and 2 V, respectively. To prevent the consolidated powder from being sintered to the die walls and plungers, as well as to easily extract the resulting compound, 200 µm thick graphite paper was used. The die was wrapped in a thermal insulating fabric to reduce the heat loss when heated. The temperature of the process was controlled by an optical pyrometer focused on a hole (5.5 mm deep) located in the middle of the die’s outer wall. In order to remove the templates, the samples were annealed in air at 800 °C for 1 h at a heating rate of 5 °C/min, in the “Nabertherm GmbH” furnace (Lilienthal, Germany).

### 2.3. Microbiological Test

Microbiological studies were done to assess the formation of *Pseudomonas aeruginosa* biofilms on the obtained samples of ceramic wollastonite. The samples were placed on the surface of the solid medium and pre-seeded with the reference strain *Pseudomonas aeruginosa.* Cultivation was carried out at 37 °C for 48 h. The biofilm was fixed on the sample by rinsing with 4% formaldehyde in a 1% solution of the phosphate buffer followed by exposure of 1% of the osmium tetroxide solution for 1 h. Dehydration was carried out with consistent treatment in ethanol of varying concentrations and at appropriate exposures (30%—10 min; 50%—10 min; 70%—10 min; and 96%—20 min), and then in acetone for 20 min. The morphology of the biofilms was studied using electron microscopy.

### 2.4. Characterization Methods

The identification of crystalline phases in the original powders and the ceramics derived from them was carried out using X-ray diffraction (XRD) with CuKα-radiation (Ni-filter, average wavelength (λ) 1.5418 Å, range of angles of 10–80°, scan step 0.02°, and spectrum registration speed—5°/min) on a multipurpose X-ray diffractometer D8 Advance “Bruker AXS” (Karlsruhe, Germany). XRD patterns were taken from the Powder Diffraction File^™^ (PDF, Soorya N Kabekkodu, 2007). The reference numbers from the PDF database are as follows: Wollastonite—01-084-0654 (C) Wollastonite 1A—CaSiO_3_; hydroxyapatite: 01-074-0566 (C) Hydroxyapatite—Ca_10_(PO_4_)_6_(OH)_2_. Thermogravimetric analysis (TGA) was carried out on the derivativograph Q-1500 of the F. Paulik, J. Paulik, L. Erdey (Hungary) system in air in a platinum crucible at a heating rate of 10°/min to a maximum temperature of 1000 °C. Low-temperature nitrogen sorption was employed to determine the specific surface area (S_spec._) on an automated physisorption analyzer, Autosorb IQ “Quantachrome” (Boynton Beach, FL, USA), using the Brunauer–Emmett–Teller (BET) model. The pore size distribution was analyzed on a mercury porosimeter AutoPore IV “Micromeritics GmbH” (Norcross, GA, USA). The material’s structure and biofilm’s morphology were studied by the method of scanning electron microscopy (SEM) on an Ultra 55 “Carl Zeiss” (Oberkochen, Germany) with a field-emission cathode and Oxford X-Max detector for Energy-dispersive X-ray (EDX) spectroscopy. The composite ceramic samples were covered by 5 nm of platinum and were investigated at the fracture sites at accelerating voltages of 5 kV and at 20 kV for EDX analysis. To minimize irradiation and to exclude the samples charging, biofilms were investigated at an accelerating voltage of 1 kV after fixing on the samples (see Section 2.3). The beam current was I ≈ 100 pA. Vickers hardness (HV) was measured at a HV0.5 load on a hardness tester, the HMV-G-FA-D “Shimadzu” (Kyoto, Japan) micro-solid. Prior to microhardness tests, the ceramic surface was polished on an automated polishing machine MECATECH 234 (Grenoble, France). Compressive strength (σ_cs_) of the cylindrically shaped samples (diameter 15.3 mm and 3–6 mm high) was determined by squeezing at a rate of 0.5 mm/min on the tensile machine Autograph AG-X plus 100 kN “Shimadzu” (Kyoto, Japan). The experimental density (ED) of the samples was determined by hydrostatic weighing on the balance Adventurer™ “OHAUS Corporation” (Parsippany, NJ, USA)

Young’s modulus (E) was evaluated in the load range of 3000–5000 N according to the following formula:

$$E=\frac{F\times h}{S\times \left(l_{2}-l_{1}\right)}$$

where F—load applied onto the sample (N); h—sample’s height (mm); S—sample’s surface area (mm^2^); and l_1_ and l_2_—starting and final height of the material (mm) in the range of applied loads.

Sample deformation along the height under applied pressure spanning 3000–5000 N was evaluated via the formula:

$$\mathrm{Deformation}\;(\%)=100-\left(\frac{l_{1}}{l_{2}}\times 100\right)$$

where *l*_1_ and *l*_2_ is the starting and final sample height (mm) in the range of applied loads.

The elative density (RD) of the composite ceramic samples was carried out according to the formula:

RD(%)=100ω1ρ1+ω2ρ2

where ω—mass content of component; and ρ—theoretical density of the component.

To calculate the porosity of the samples the formula was used:

Porosity(%)=100−(EDRD×100)

ED—experimental density, RD—relative density.

The experimental density and Vickers microhardness were obtained as the mean values out of three measurements for each sample. Determination of the compressive strength is a destructive method of analysis, so only a single measurement was done on each sample. Young’s modulus was calculated in the range of 3000–5000 N from the trends, obtained from the compressive strength measurements.

## 3. Results and Discussion 

**Figure 1:**
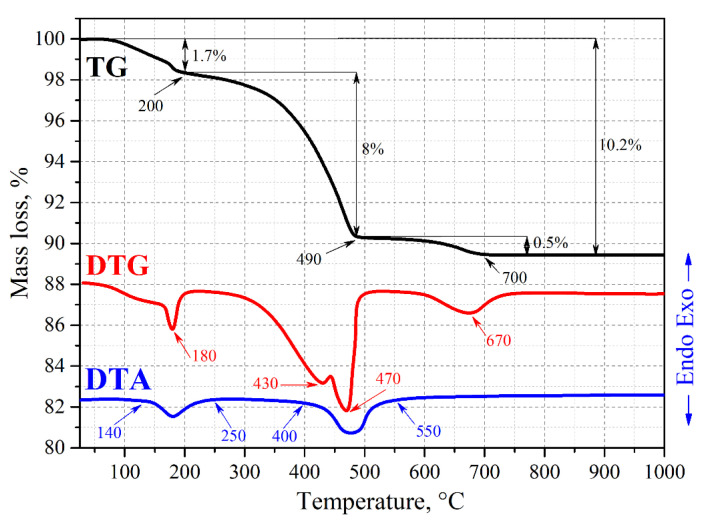
Thermogram of the reaction mixture (RM) sample heated in air.

Primarily, the chemical interaction between the components of the reaction mixture (RM) was studied to reveal the HAp formation according to Reaction (2) in air as well as to simulate the conditions of SPS consolidation in the presence of the hydrated calcium silicate xerogel (xonotlite). The temperature when the RM components started to react (Reaction (2)) was determined by thermogravimetric analysis (Figure 1).

According to the results of the TGA (Figure 1), a slight mass loss of 1.7 wt% is observed till 200 °C on the TG and DTG curves, while an endo-effect associated with it lasts till 250 °C on DTA. A further increase in temperature leads to the 8 wt% weight loss by 490 °C on TG due to the solid-phase interaction of the RM components yielding HAp and water by Reaction (2) with the onset of the endo-effect being observed from 400 to 550 °C on the DTA curve. Moreover, at these temperatures the non-reacted calcium hydrophosphate is likely to decompose according to Reaction (3).

2CaHPO_4_ = Ca_2_P_2_O_7_ + H_2_O (3)

There is a 0.5% weight loss at 700 °C on the TG curve with a minimum at 670 °C on DTG, which can be caused by the partial decomposition of calcium carbonate according to Reaction (4).

CaCO_3_ = CaO + CO_2_↑ (4)

**Figure 2:**
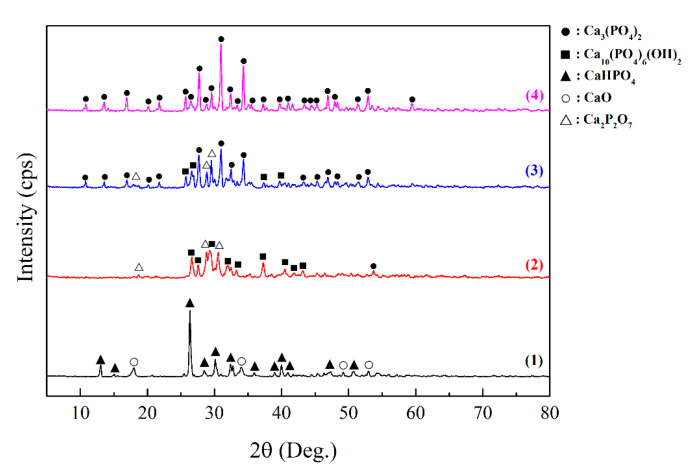
The XRD patterns of the RM powder in its original form (**1**) and after its heat treatment in air at the following temperatures: (**2**) 500–700 °C, (**3**) 900 °C, and (**4**) 1000 °C.

To confirm the results of the TGA, XRD was performed for the RM samples before and after annealing in air at different temperatures (Figure 2).

The RM composition consists of the CaO and CaHPO_4_ crystalline phases (Figure 2, Pattern 1). Thermo-oxidative annealing initiates a solid phase reaction of the RM with the formation of HAp at the temperature ranging from 500 to 700 °C (Figure 2, Pattern 2) according to Reaction (2), which was also noted on TGA (Figure 1). An impurity of calcium pyrophosphate (Ca_2_P_2_O_7_), which can be formed according to Reaction (3), has been identified in this sample. 

An increased annealing temperature leads to partial (at 900 °C) and complete (at 1000 °C) decomposition of HAp with the formation of calcium orthophosphate (Ca_3_(PO_4_)_2_) (Figure 2, Pattern 3) according to Reaction (5):

Ca_10_(PO_4_)_6_(OH)_2_ = 3Ca_3_(PO_4_)_2_ + CaO + H_2_O. (5)

**Figure 3:**
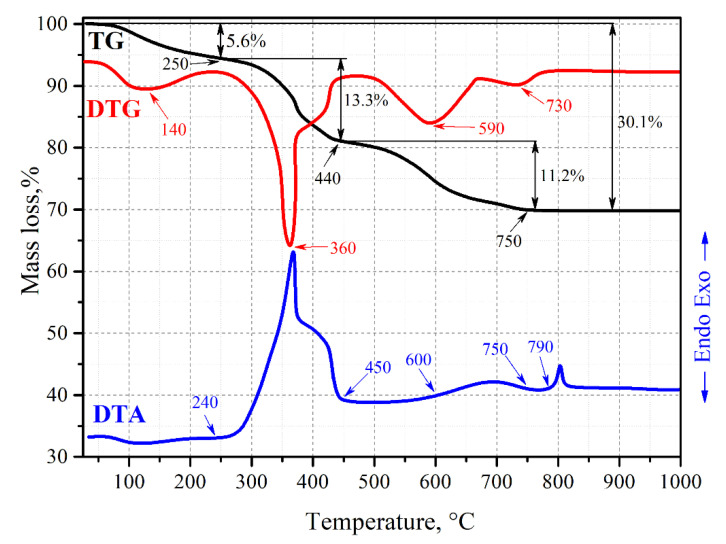
Thermograms of sintering mixture (SM) sample containing 20 wt% of the RM.

**Figure 4:**
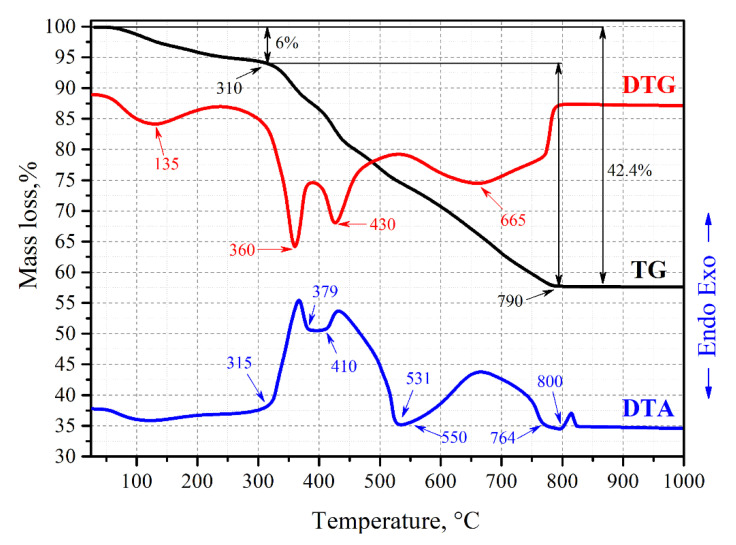
Thermograms of the SM sample containing 20 wt% of the RM as well as 10 wt% of carbon fiber and 10 wt% of graphite powder.

Then, we investigated formation of wollastonite combined with HAp from the sintering mixture (SM), which consists of a xerogel (Ca_6_Si_6_O_17_·2OH (xonotlite)) obtained via sol-gel and an RM along with the pore-forming templates (latex, carbon fiber, and graphite powder). To do this, the TGA was implemented and the corresponding thermograms (Figure 3 and Figure 4) were obtained. According to them, the mass loss of 5.6 wt% in the samples by 250 °C on TG is mainly due to partial dehydration of xonotlite (Figure 3). When the temperature rises, the pore-forming agent, organo-silicon latex, gets oxidized and decomposed. This process results in a loss of sample’s mass of 13.3 wt% and characterized by an exo-effect within 240–450 °C on DTA and a minimum at 360 °C on the DTG. Up to 450 °C, the dehydration of the xonotlite is complete and the latex becomes partially burnt out from the bulk. There is also a solid-phase interaction between the RM components in this temperature range, yielding HAp as it was studied above (Figure 1) according to Reaction (2). The remaining latex part is burnt out by 750 °C on the DTA along with the associated minima observed at 590 and 730 °C on DTG, resulting in a mass loss of 11.2 wt%. Partial HAp decomposition may also take place according to Reaction (4). The total weight loss was 30.1 wt%. The exo-effect with an inflection point at 790 °C on DTA is caused by the crystallization of the wollastonite.

The initial stage of the SM sample annealing in the presence of carbon pore-formers manifests water removal with a mass loss of about 6 wt% by 310 °C on the TG curve (Figure 4), which is similar to the above sample. However, further heating of the sample has a significant difference associated with the imposed templates. Along with latex burnout from the xonotlite’s bulk, the exo-effect at 315–450 °C on DTA merges with the exo-effect coming from the graphite powder burning within 410–531 °C. Decomposition of another template, carbon fiber, takes place in the range 550–764 °C on DTA. These effects correspond to the minima on the DTG at 360, 430, and 665 °C, related to the decomposition of latex, graphite powder, and carbon fibers, respectively. Besides, that region also encompasses latex burnout by 750 °C on TG, as was shown earlier on Figure 3. The decomposition processes of the carbon fiber and graphite powder occur simultaneously, causing a large area of exo-effect on the DTA curve in the range 531–764 °C. The formation of crystalline wollastonite is in this case similar to the above-described pattern without carbon additives, which occurs with the onset of heat at 800 °C on the DTA. It is worth noticing the continuous nature of the mass loss in this sample reaching 42.4 wt% by 790 °C on the TG curve, which is significantly higher in comparison with the sample without the carbon additives discussed above. This difference is caused by the thermo-oxidative destruction of the carbon pore-forming agents.

**Figure 5:**
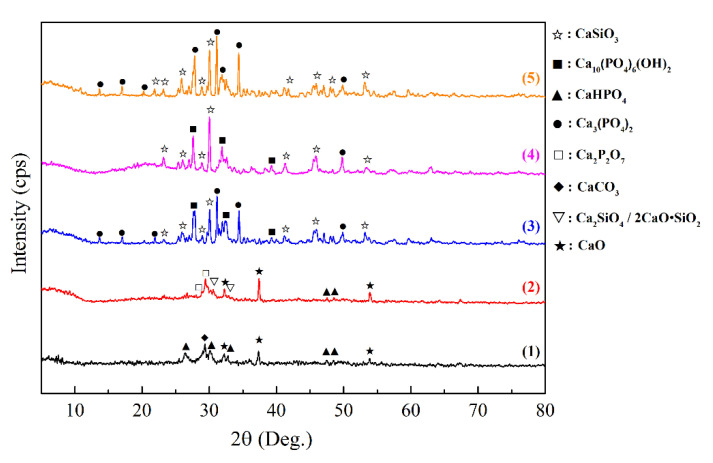
Thermograms of the original SM sample containing 20 wt% of the RM (**1**), as well as its derivatives annealed at different temperatures: (**2**)—500 °C; (**3**)—900 °C; (**4**)—900 °C, the sample containing pore-forming agents (10 wt% CF and 10 wt% GP); and (**5**)—1000 °C.

According to the XRD, the composition of the original SM sample includes the phases of the RM components (CaO and CaHPO_4_), while the silicate phase cannot be found, evidencing its amorphous structure (Figure 5, Pattern 1). After annealing at 500 °C, the phase of calcium orthosilicate Ca_2_SiO_4_ is observed, which indicates the intermediate stage of crystalline wollastonite formation (Figure 5, Pattern 2). The presence of calcium silicate in this mixture slows down the interaction between the RM components at this temperature, as there is no HAp phase in the material. Annealing at 900 °C leads to the formation of crystalline wollastonite as well as HAp as a result of the interaction between the RM components (Figure 5, Pattern 3). In addition, there is an impurity of calcium orthophosphate Ca_3_(PO_4_)_2_ caused by the HAp decomposition according to Reaction (5). Annealing of the SM sample containing carbon pore-forming agents at the same temperature (900 °C) also yields wollastonite and HAp (Figure 5, Pattern 4) without formation of calcium orthophosphate, which is likely caused by HAp phase stabilization during templates oxidation.

According to the described results, the optimal temperature to obtain crystalline wollastonite and to initiate an “in situ” reaction between the RM components for the formation of the HAp phase is found to be 900 °C. In addition, the temperature of SPS below 900 °C is not enough for sintering of wollastonite, because the required mechanical characteristics are not achieved as was shown in our previous study [44]. 

**Figure 6:**
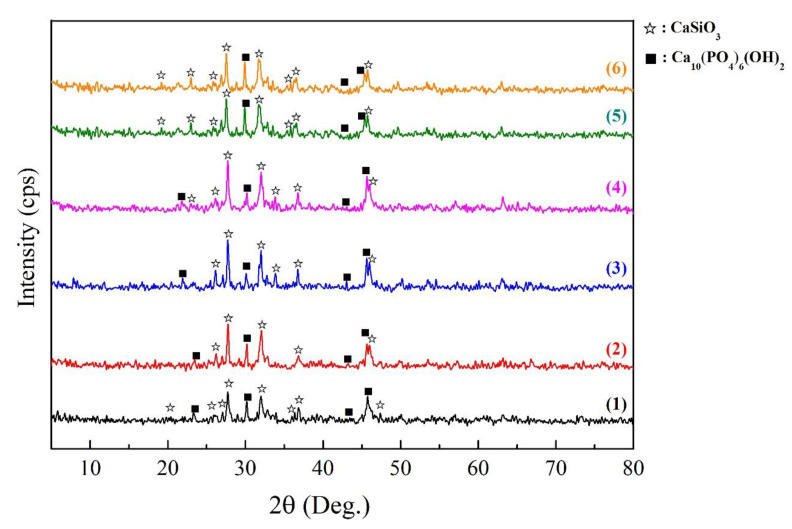
XRD patterns of the composite ceramics obtained by SPS-RS at 900 °C and their subsequent thermo-oxidative treatment at 800 °C: (**1**)—CaSiO_3_-(50)HAp; (**2**)—CaSiO_3_-(20)HAp; (**3**)—CaSiO_3_-(20)Hap/5(Cw); (**4**)—CaSiO_3_-(20)HAp/10(Cw); (**5**)—CaSiO_3_-(20)HAp/5(Cw)-10(Cp); and (**6**)—CaSiO_3_-(20)HAp/10(Cw)-10(Cp). The sample description is presented in Table 1.

XRD patterns of the SPS ceramic samples obtained from the SM (Figure 6) shows the phase composition corresponding to a composite based on crystalline wollastonite and HAp. For all samples, the composition is identical regardless of the HAp content and the type of pore-forming template used.

**Figure 7:**
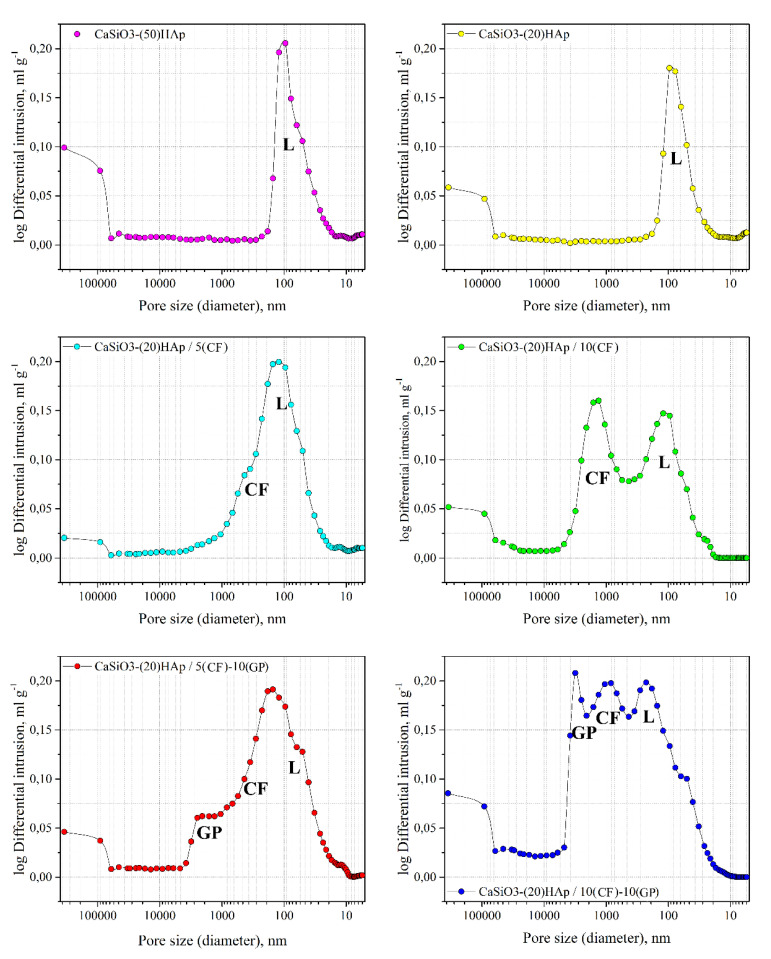
Differential mercury intrusion curves obtained for the porous SPS composite ceramics. Sample denotation is presented in Table 1, the structural characteristics are in Table 2. The indexes (GP, CF, and L) show the pore size ranges tailored by certain types of pore-forming agents (templates): L—siloxane-acrylate latex, CF—carbon fiber, and GP—graphite powder.

The results of porosimetry and structural analysis revealed the effect of the templates on the structure of the obtained ceramics samples. Differential mercury intrusion analysis shows the average size of the macropores provided by the polymer latex is about 100 nm (Figure 7). Changing the amount of HAp in the ceramics from 20 to 50 wt% does not lead to significant structural changes, with the pore size distribution being maintained in the specified interval (samples CaSiO_3_—(20)HAp and CaSiO_3_—(50)HAp). The surface area for these samples, calculated by BET, lies in the same range of 2.6 and 2.8 m^2^·g^−1^ (Table 2).

The introduction of carbon fiber (5 and 10 wt%) into the synthesis and its subsequent removal changes the range of the pore sizes in the resulting ceramics. When 5 wt% CF are added, the average pore size is approximately 100 nm but the base of the intrusion curve has extended, indicating the formation of larger pores sized above 100 nm (sample CaSiO_3_-(20)HAp/5(CF)). The number of these pores increases with the amount of the additive. At 10 wt% of CF, there emerges a peak at about the 1–5 µm range on the differential intrusion curve (CaSiO_3_-(20)HAp/10(CF) sample). At the same time, S_spec._ decreases from 2.9 to 1.9 m^2^·g^−1^ (Table 2), which is probably due to the exclusion of the micro and mesopores (less than 50 nm) from the sample’s structure at the moment of carbon template oxidation and macropores formation. There is an extended base of the intrusion curve observed for the sample containing 5 wt% of powder graphite (CaSiO_3_-(20)HAp/5(CF)-10(GP) sample) with a separate maximum appearing in the range above 5 µm (CaSiO_3_-(20)HAp/10(CF)-10(GP) sample). S_spec._ increases for the samples under consideration to 4.9 and 4 m^2^·g^−1^, respectively (Table 2). The general trend observed for all samples is the increase in the macropores’ volume from 0.18 to 0.43 mL/g, depending on the type and quantity of the used template as well as the growth of the total porosity in the system from 8.3 to 27.7% (Table 2).

**Figure 8:**
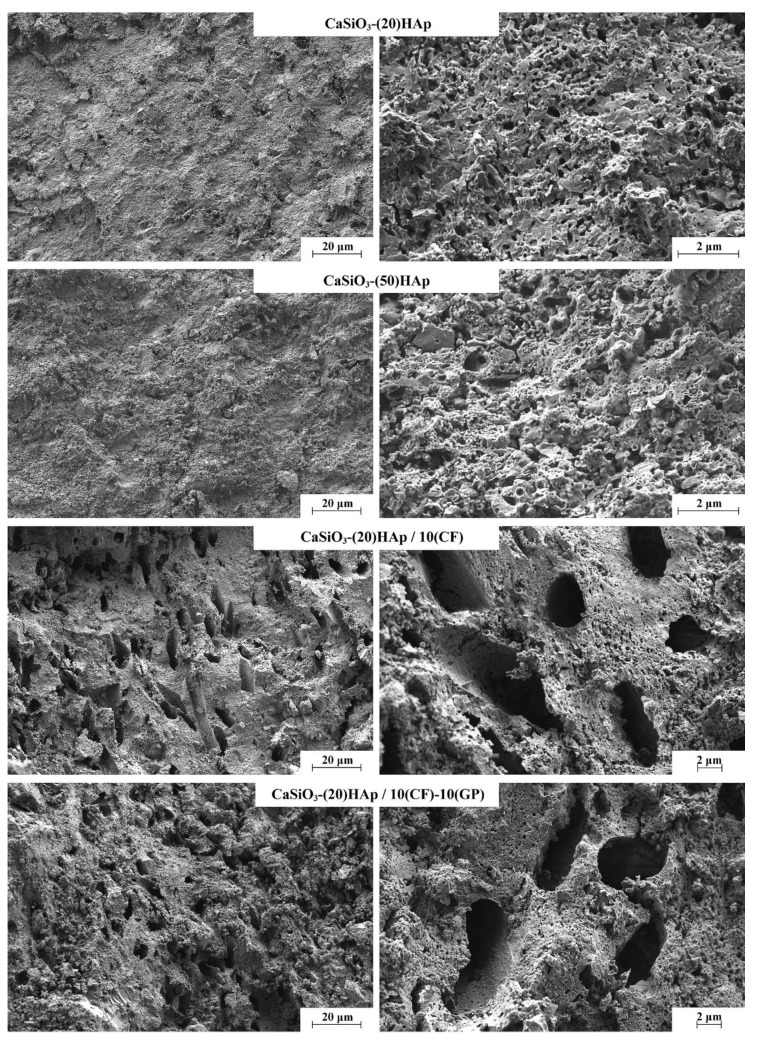
SEM images of the spark plasma sintering–reactive synthesis (SPS-RS) composite ceramics surface. The sample descriptions are presented in Table 1.

Changes in the structure of samples are confirmed by microscopic studies. SEM images (Figure 8) show that the samples synthesized with a different HAp content in the presence of a polymer latex (CaSiO_3_-(20)HAp and CaSiO_3_-(50)HAp) samples) are characterized by the presence of pores sized below 200 nm, which is commensurate to the template’s size. Additional carbon pore-forming agents produce larger pores of 1 µm and above, the shape and size of which are commensurate to that of carbon fiber as, e.g., for the CaSiO_3_-(20)HAp/10(CF) sample. The introduction of graphite powder into the synthesis leads to a distorted arrangement of the pores derived from CF with the structure becoming looser (CaSiO_3_-(20)HAp/10(CF)-10(GP)) (Figure 8). In both samples, when the large pores (larger than 1 µm) are forming, the presence of the small pores (less than 200 nm) is maintained.

**Figure 9:**
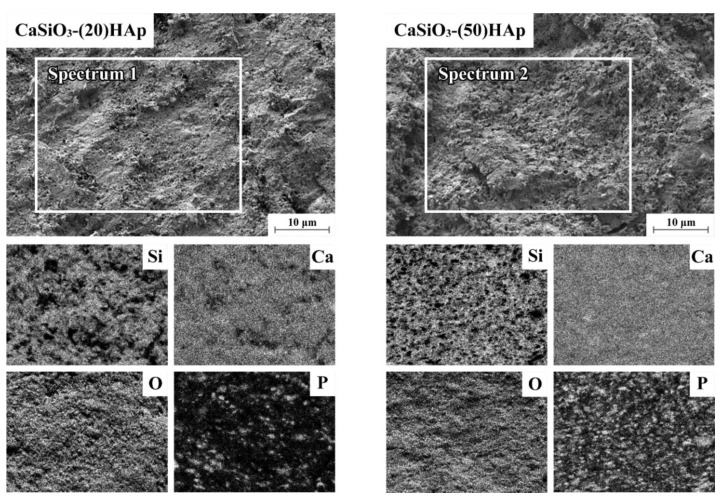
Elements mapping (EDX analysis) on the surface of the composite ceramic samples containing 20 wt% (the CaSiO3-(20)HAp sample) and 50 wt% HAp (the CaSiO3-(20)HAp sample).

Based on the EDX data, the presence of phosphorus on the surface of the samples is found, which indicates the presence of HAp in the ceramic wollastonites. The uniform distribution of phosphorus in the samples can be observed on the map of the elements, which reflects the difference when the number of HAp increases from 20 to 50 wt% (Figure 9, Table 3). According to the quantitative EDX analysis, there is also a difference in the phosphorus content of the ceramics. Analysis of arbitrary surface areas of the samples with a different HAp content (Figure 9, Spectra 1 and 2) indicates a phosphorus content of 2.15 and 4.78 wt% (Table 3). A 2.2-fold difference in phosphorous content corresponds well to the amounts of introduced RM components into the sintering mixture within the error of the method.

The physical and mechanical characteristics of the biocomposite ceramics (Table 4) were determined. The relative density (RD) of the samples with a different HAp content ranges between 90.2 and 91.7% under certain conditions of SPS consolidation. The increase in the HAp content from 20 to 50 wt% in the ceramic composite leads to an increase in compressive strength (σ_cs._) from 302 to 362 MPa and in microhardness (HV) from 134 to 146 (Table 4, Samples 1 and 2). The total porosity of the ceramics formed by polymer latex is reduced from 9.7 to 8.3% (Table 2) due to the fact that the amount of HAp in the sample is in direct relation to the original silicate raw material (xonotlite), which contains the latex template. When the ratio of xonotlite to HAp changes, the amount of latex responsible for porous structure formation thereby alters the strength characteristics of the final ceramics.

The total porosity of the system was enhanced in this work by means of additional carbon pore-forming agents (Table 2), although it significantly reduces the density and affects the mechanical characteristics of the samples (Table 4). Adding 10 wt% carbon fiber into the synthesis is found to be optimal as it yields porous (19.7%) and dense (RD 80%) biocomposite ceramics, with the mechanical strength (σ_cs._ 111 MPa) lying within the values of the normal strength of natural bone (110–120 MPa) [53,54,55]. Introducing powder carbon as an additional pore-forming agent is also useful for increasing the overall porosity of the ceramics, but its quantity should be clearly defined with the other characteristics achieved.

**Figure 10:**
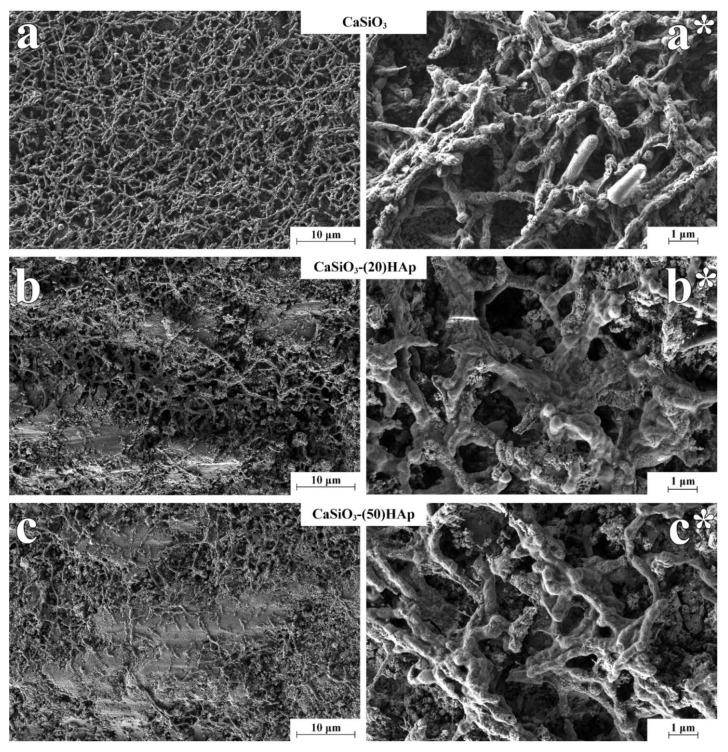
SEM images of bacterial film on ceramic samples with different HAp content obtained by SPS-RS: (**a**,a*) 0 wt% HAp—continuous layer of bacterial cells without an alginate layer; (**b**,b*) 20 wt% HAp—less dense layer of bacterial cells coated with an alginate layer; (**c**,c*) 50 wt% HAp—rarefied layer of bacterial cells coated with a dense layer of alginate.

The work carried out a microbiological test to study the formation and morphological features of bacterial film (biofilm) based on *Pseudomonas aeruginosa* on the surface of composite ceramic samples with different HAp contents. In addition, a sample of pristine ceramic wollastonite without the HAp additive, which was obtained in our earlier studies, was used for comparison [42]. The type of bacteria implemented here lives in the external environment (water and soil) and is one of the leading pathogens that cause post-surgery infections requiring medical care [56]. According to the SEM images (Figure 10), all the samples clearly show that a mature biofilm had formed. On the sample of ceramic wollastonite without HAp, the biofilm is distributed in a denser, uniform layer (Figure 10a). On the contrary, in the case of composite ceramics, the monolithic agglomerates of the HAp phase are less intensely populated by bacteria with the latter occupying mostly places of porous and loose formations (Figure 10b,c). All samples between the cells clearly show cytoplasmic bridges, which is a sign of an actively developing biofilm. The main point to be noted is that the bacteria grown on the samples containing HAp are covered with a thick layer of alginate, which indicates the inclusion of a protective reaction of the bacteria under the negative impact of the environment caused by the presence of HAp in the ceramics and the completion of the biofilm formation (Figure 10b*,c*). The formation of an alginate layer indicates that the presence of HAp in the ceramic prevented the formation of a dense uniform (continuous) biofilm layer. In this regard, in terms of the risk of infection, the composite HAp-containing ceramics contained is more attractive for biomedical applications.

## 4. Conclusions

The work provides an original way to obtain CaSiO_3_-HAp porous ceramics with the wet and solid-phase synthesis strategies being combined. The method involves sol-gel synthesis (template) of the starting raw material in the form of an amorphous composite material based on xonotlite and its subsequent spark plasma sintering yielding ceramic wollastonite. TGA, XRD, and EDX showed that the HAp phase formation in the resulting ceramics can be initiated by the solid-phase interaction of the reaction mixture (CaO and CaHPO_4_) in the spark plasma heating by the “in situ” reaction directly at the moment of sintering of the amorphous xonotlite. It has been determined that the optimal temperature for the SPS-RS to obtain crystalline wollastonite from the amorphous xonotlite, as well as to initiate the reaction “in situ” with the formation of HAp, is 900 °C. 

Nitrogen physisorption and mercury porosimetry allowed to reveal that the pore size and volume depend on the type and quantity of the template introduced at the different stages of the synthesis, leaving pores after the thermal-oxidative treatment of the ceramics. It is shown that latex introduced in the sol-gel synthesis allows to form pores of about 100 nm, while carbon pore-forming agents added prior to SPS-RS contribute to the formation of macropores of 1 µm and larger. We determined that the use of latex and 10 wt% carbon fiber is considered the optima, as porous (19.7%) and dense (RD 80%) biocomposite ceramics is formed that exhibit the required mechanical strength (σ_cs._ 111 MPa), which is within the values of the normal strength of natural bone (110–120 MPa). The use of graphite powder should be carefully adjusted due to its impact on the other important physical and mechanical characteristics of ceramics. 

The results of the microbiological test revealed that the (20 and 50 wt%) HAp-containing ceramic samples show the bacteria *Pseudomonas aeruginosa* being covered with a thick layer of alginate, with the latter not being observed on pristine wollastonite. Alginate appearance indicates the protective response of the bacteria to a negative environmental impact. In this regard, in terms of the risk of infection, composite HAp-containing ceramics is more attractive for biomedical applications.

## Figures and Tables

**Table 1:** Wollastonite/HAp and the amounts of added templates.

#	Sample Notation	Ratio, wt%		Quantity, wt%	
		Wollastonite	* HAp	** Carbon Fiber (CF)	** Graphite Powder (GP)
1	CaSiO_3_-(50)Hap	50	50	-	-
2	CaSiO_3_-(20)Hap	80	20	-	-
3	CaSiO_3_-(20)Hap/5(CF)	80	20	5	-
4	CaSiO_3_-(20)Hap/10(CF)	80	20	10	-
5	CaSiO_3_-(20)Hap/5(CF)-10(GP)	80	20	5	10
6	CaSiO_3_-(20)Hap/10(CF)-10(GP)	80	20	10	10

Note: * Estimated amount of HAp according to the reaction Equation (2); ** weight amount in terms of the total mass of the sintering mixture.

**Table 2:** Structural characteristics of the composite ceramics samples.

#	Sample	S_spec._, m^2^·g^−1^	V_pore_, mL/g	Porosity, %
1	CaSiO_3_-(50)HAp	2.8 ± 0.1	0.18 ± 0.01	8.3
2	CaSiO_3_-(20)HAp	2.6 ± 0.1	0.13 ± 0.01	9.7
3	CaSiO_3_-(20)HAp/5(CF)	2.9 ± 0.1	0.21 ± 0.01	18.5
4	CaSiO_3_-(20)HAp/10(CF)	1.9 ± 0.1	0.26 ± 0.01	19.7
5	CaSiO_3_-(20)HAp/5(CF)-10(GP)	4.9 ± 0.1	0.28 ± 0.01	25.4
6	CaSiO_3_-(20)HAp/10(CF)-10(GP)	4 ± 0.1	0.43 ± 0.01	27.7

**Table 3:** The quantitative surface elemental composition (EDX analysis) of the composite ceramic samples presented in Figure 9 (wt%).

Samples	Spectra	C	O	Si	P	Ca
CaSiO_3_-(20)HAp	Spectrum 1	6.31 ± 0.03	67.49 ± 0.03	11.84 ± 0.03	2.15 ± 0.03	12.21 ± 0.03
CaSiO_3_-(50)HAp	Spectrum 2	6.96 ± 0.03	68.81 ± 0.03	8.09 ± 0.03	4.78 ± 0.03	11.36 ± 0.03

**Table 4:** Physical and mechanical characteristics of the composite ceramics samples.

#	Samples	ED, g·sm^−3^	RD, %	HV	σ_cs._, MPa	E, MPa (3000–5000 H)	Deformation, %
1	CaSiO_3_-(50)HAp	2.723 ± 0.005	91.7	146	362	2564	5.3
2	CaSiO_3_-(20)HAp	2.586 ± 0.005	90.2	134	302	2224	4.1
3	CaSiO_3_-(20)HAp/5(CF)	2.334 ± 0.005	81.4	64	176	1997	5.2
4	CaSiO_3_-(20)HAp/10(CF)	2.301 ± 0.005	80	34	111	1421	6.3
5	CaSiO_3_-(20)HAp/5(CF)-10(GP)	2.137 ± 0.005	74.6	26	75	1379	8.7
6	CaSiO_3_-(20)HAp/10(CF)-10(GP)	2.069 ± 0.005	72.2	1<	35	1358	9.2

## References

[1] Dubok V.A. (2001). Bioceramics—Yesterday, Today, Tomorrow. Powder Metall. Met. Ceram..

[2] Stevens M.M. (2008). Biomaterials for bone materials that enhance bone regeneration have a wealth of potential. Bone.

[3] Sainitya R., Sriram M., Kalyanaraman V., Dhivya S., Saravanan S., Vairamani M., Sastry T.P., Selvamurugan N. (2015). Scaffolds containing chitosan/carboxymethyl cellulose/mesoporous wollastonite for bone tissue engineering. Int. J. Biol. Macromol..

[4] Sautier J.M., Kokubo T., Ohtsuki T., Nefussi J.R., Boulekbache H., Oboeuf M., Loty S., Loty C., Forest N. (1994). Bioactive glass-ceramic containing crystalline apatite and wollastonite initiates biomineralization in bone cell cultures. Calcif. Tissue Int..

[5] Saadaldin S.A., Rizkalla A.S. (2014). Synthesis and characterization of wollastonite glass-ceramics for dental implant applications. Dent. Mater..

[6] Papynov E.K., Shichalin O.O., Apanasevich V.I., Portnyagin A.S., Yu M.V., Yu B.I., Merkulov E.B., Kaidalova T.A., Modin E.B., Afonin I.S. (2020). Sol-gel (template) synthesis of osteoplastic CaSiO_3_/HAp powder biocomposite: “In vitro” and “in vivo” biocompatibility assessment. Powder Technol..

[7] Bheemaneni G., Saravana S., Kandaswamy R. (2018). Processing and characterization of poly(butylene adipate-co-terephthalate)/wollastonite biocomposites for medical applications. Mater. Today Proc..

[8] Maxim L.D., Niebo R., Utell M.J., McConnell E.E., Larosa S., Segrave A.M. (2014). Wollastonite toxicity: An update. Inhal. Toxicol..

[9] Lin K., Lin C., Zeng Y. (2016). High mechanical strength bioactive wollastonite bioceramics sintered from nanofibers. RSC Adv..

[10] Hu Y., Xiao Z., Wang H., Ye C., Wu Y., Xu S. (2018). Fabrication and characterization of porous CaSiO_3_ ceramics. Ceram. Int..

[11] Adams L.A., Essien E.R., Kaufmann E.E. (2018). A new route to sol-gel crystalline wollastonite bioceramic. J. Asian Ceram. Soc..

[12] Tian L., Wang L., Wang K., Zhang Y., Liang J. (2019). The Preparation and properties of porous sepiolite ceramics. Sci. Rep..

[13] Biswas N., Samanta A., Podder S., Ghosh C.K., Ghosh J., Das M., Mallik A.K., Mukhopadhyay A.K. (2018). Phase pure, high hardness, biocompatible calcium silicates with excellent anti-bacterial and biofilm inhibition efficacies for endodontic and orthopaedic applications. J. Mech. Behav. Biomed. Mater..

[14] Ingole V.H., Sathe B., Ghule A.V. (2018). Bioactive ceramic composite material stability, characterization, and bonding to bone. Fundamental Biomaterials: Ceramics.

[15] Ni S., Chang J. (2009). In vitro degradation, bioactivity, and cytocompatibility of calcium silicate, dimagnesium silicate, and tricalcium phosphate bioceramics. J. Biomater. Appl..

[16] De Aza P.N., Guitian F., De Aza S. (1994). Bioactivity of wollastonite ceramics: In vitro evaluation. Scr. Metall. Mater..

[17] Azarov G.M., Maiorova E.V., Oborina M.A., Belyakov A.V. (1995). Wollastonite raw materials and their applications (a review). Glas. Ceram..

[18] Golovanova O.A. (2018). Experimental Modeling of Formation of the Basic Mineral Phases of Calcifications. Russ. J. Inorg. Chem..

[19] Fadeeva T.V., Golovanova O.A. (2019). Physicochemical Properties of Brushite and Hydroxyapatite Prepared in the Presence of Chitin and Chitosan. Russ. J. Inorg. Chem..

[20] Huang Q.W., Wang L.P., Wang J.Y. (2014). Mechanical properties of artificial materials for bone repair. J. Shanghai Jiaotong Univ..

[21] Juhasz J.A., Best S.M. (2012). Bioactive ceramics: Processing, structures and properties. J. Mater. Sci..

[22] Guglielmi M., Kickelbick G., Martucci A., Aegerter M., Prassas M. (2014). Sol-Gel Nanocomposites.

[23] Ros-Tárraga P., Murciano Á., Mazón P., Gehrke S.A., De Aza P.N. (2017). In vitro behaviour of sol-gel interconnected porous scaffolds of doped wollastonite. Ceram. Int..

[24] Lin K., Chang J., Chen G., Ruan M., Ning C. (2007). A simple method to synthesize single-crystalline β-wollastonite nanowires. J. Cryst. Growth.

[25] Singh N.B. (2006). Hydrothermal synthesis of β-dicalcium silicate (β-Ca_2_SiO_4_). Prog. Cryst. Growth Charact. Mater..

[26] Solonenko A.P., Blesman A.I., Polonyankin D.A. (2018). Preparation and in vitro apatite-forming ability of hydroxyapatite and β-wollastonite composite materials. Ceram. Int..

[27] Palakurthy S., Samudrala R.K. (2019). In vitro bioactivity and degradation behaviour of β-wollastonite derived from natural waste. Mater. Sci. Eng. C.

[28] Ismail H., Shamsudin R., Abdul Hamid M.A. (2016). Effect of autoclaving and sintering on the formation of β-wollastonite. Mater. Sci. Eng. C.

[29] Bakan F., Laçin O. (2013). A novel low temperature sol—gel synthesis process for thermally stable nano crystalline hydroxyapatite. Powder Technol..

[30] Wang P., Li C., Gong H., Jiang X., Wang H., Li K. (2010). Effects of synthesis conditions on the morphology of hydroxyapatite nanoparticles produced by wet chemical process. Powder Technol..

[31] Xu J.L., Khor K.A., Kumar R. (2007). Physicochemical differences after densifying radio frequency plasma sprayed hydroxyapatite powders using spark plasma and conventional sintering techniques. Mater. Sci. Eng. A.

[32] Ergun C. (2011). Enhanced phase stability in hydroxylapatite/zirconia composites with hot isostatic pressing. Ceram. Int..

[33] Viswanathan V., Laha T., Balani K., Agarwal A., Seal S. (2006). Challenges and advances in nanocomposite processing techniques. Mater. Sci. Eng. R Rep..

[34] Orru R., Licheri R., Locci A.M., Cincotti A., Cao G. (2009). Consolidation/synthesis of materials by electric current activated/assisted sintering. Mater. Sci. Eng. R Rep..

[35] Tokita M., Somiya S. (2013). Spark Plasma Sintering (SPS) Method, Systems, and Applications. Handbook of Advanced Ceramics: Materials, Applications, Processing and Properties.

[36] Simonenko T.L., Kalinina M.V., Simonenko N.P., Simonenko E.P., Glumov O.V., Mel’nikova N.A., Murin I.V., Shichalin O.O., Papynov E.K., Shilova O.A. (2018). Spark plasma sintering of nanopowders in the CeO_2_-Y_2_O_3_ system as a promising approach to the creation of nanocrystalline intermediate-temperature solid electrolytes. Ceram. Int..

[37] Simonenko E.P., Simonenko N.P., Simonenko T.L., Grishin A.V., Tal’skikh K.Y., Gridasova E.A., Papynov E.K., Shichalin O.O., Sevastyanov V.G., Kuznetsov N.T. (2019). Sol-gel synthesis of SiC@Y_3_Al_5_O_12_ composite nanopowder and preparation of porous SiC-ceramics derived from it. Mater. Chem. Phys..

[38] Papynov E.K., Shichalin O.O., Mayorov V.Y., Modin E.B., Portnyagin A.S., Tkachenko I.A., Belov A.A., Gridasova E.A., Tananaev I.G., Avramenko V.A. (2017). Spark Plasma Sintering as a high-tech approach in a new generation of synthesis of nanostructured functional ceramics. Nanotechnol. Russ..

[39] Wang H., He Z., Li D., Lei R., Chen J., Xu S. (2014). Low temperature sintering and microwave dielectric properties of CaSiO_3_–Al_2_O_3_ ceramics for LTCC applications. Ceram. Int..

[40] Chen S., Zhou X., Zhang S., Li B., Zhang T. (2010). Low temperature preparation of the β-CaSiO_3_ ceramics based on the system CaO-SiO_2_-BaO-B_2_O_3_. J. Alloys Compd..

[41] Harabi A., Chehlatt S. (2013). Preparation process of a highly resistant wollastonite bioceramics using local raw materials. J. Therm. Anal. Calorim..

[42] Papynov E.K., Shichalin O.O., Mayorov V.Y., Modin E.B., Portnyagin A.S., Gridasova E.A., Agafonova I.G., Zakirova A.E., Tananaev I.G., Avramenko V.A. (2017). Sol-gel and SPS combined synthesis of highly porous wollastonite ceramic materials with immobilized Au-NPs. Ceram. Int..

[43] Papynov E.K., Shichalin O.O., Modin E.B., Mayorov V.Y., Portnyagin A.S., Kobylyakov S.P., Golub A.V., Medkov M.A., Tananaev I.G., Avramenko V.A. (2016). Wollastonite ceramics with bimodal porous structures prepared by sol–gel and SPS techniques. RSC Adv..

[44] Papynov E.K., Mayorov V.Y., Portnyagin A.S., Shichalin O.O., Kobylyakovt S.P., Kaidalova T.A., Nepomnyashiy A.V., Sokol’nitskaya T.A., Zub Y.L., Avramenko V.A. (2015). Application of carbonaceous template for porous structure control of ceramic composites based on synthetic wollastonite obtained via Spark Plasma Sintering. Ceram. Int..

[45] Papynov E.K., Shichalin O.O., Buravlev I.Y., Portnyagin A.S. (2020). Reactive spark plasma synthesis of porous bioceramic wollastonite. Russ. J. Inorg. Chem..

[46] Papynov E.K., Shichalin O.O., Apanasevich V.I., Afonin I.S., Evdokimov I.O., Mayorov V.Y., Portnyagin A.S., Agafonova I.G., Skurikhina Y.E., Medkov M.A. (2019). Synthetic CaSiO_3_ sol-gel powder and SPS ceramic derivatives: “In vivo” toxicity assessment. Prog. Nat. Sci. Mater. Int..

[47] Dudina D.V., Mukherjee A.K. (2013). Reactive spark plasma sintering: Successes and challenges of nanomaterial synthesis. J. Nanomater..

[48] Simonenko E.P., Simonenko N.P., Papynov E.K., Shichalin O.O., Golub A.V., Mayorov V.Y., Avramenko V.A., Sevastyanov V.G., Kuznetsov N.T. (2017). Preparation of porous SiC-ceramics by sol–gel and spark plasma sintering. J. Sol-Gel Sci. Technol..

[49] Kosyanov D.Y., Yavetskiy R.P., Vorona I.O., Shichalin O.O., Papynov E.K., Vornovskikh A.A., Kuryavyi V.G., Vovna V.I., Golokhvast K.S., Tolmachev A.V. (2017). Transparent 4 at% Nd^3+^:Y_3_Al_5_O_12_ ceramic by reactive spark plasma sintering. AIP Conf. Proc..

[50] Papynov E.K., Shichalin O.O., Skurikhina Y.E., Turkutyukov V.B., Medkov M.A., Grishchenko D.N., Portnyagin A.S., Merkulov E.B., Apanasevich V.I., Geltser B.I. (2019). ZrO_2_-phosphates porous ceramic obtained via SPS-RS “in situ” technique: Bacteria test assessment. Ceram. Int..

[51] Wang H.H., Li X.R., Fei G.Q., Mou J. (2010). Synthesis, morphology and rheology of core-shell silicone acrylic emulsion stabilized with polymerisable surfactant. Express Polym. Lett..

[52] Papynov E.K., Mayorov V.Y., Palamarchuk M.S., Bratskaya S.Y., Avramenko V.A. (2013). Sol–gel synthesis of porous inorganic materials using “core–shell” latex particles as templates. J. Sol-Gel Sci. Technol..

[53] Suchanek W., Yoshimura M. (1998). Processing and properties of hydroxyapatite-based biomaterials for use as hard tissue replacement implants. J. Mater. Res..

[54] Ravaglioli A., Krajewski A. (1992). Bioceramics.

[55] Peppas N.A., Black J., Hastings G. (1998). Handbook of Biomaterial Properties.

[56] Callejas-Díaz A., Fernández-Pérez C., Ramos-Martínez A., Múñez-Rubio E., Sánchez-Romero I., Vargas Núñez J.A. (2018). Impact of Pseudomonas aeruginosa bacteraemia in a tertiary hospital: Mortality and prognostic factors. Med. Clin..

